# Natural, Everyday Language Use Provides a Window into the Integrity of Older Adults’ Cognitive Functioning

**DOI:** 10.1093/geroni/igaa057.2123

**Published:** 2020-12-16

**Authors:** Angelina Polsinelli, Suzanne Moseley, Matthew Grilli, Elizabeth Glisky, Matthias Mehl

**Affiliations:** 1 Indiana University School of Medicine, Indianapolis, Indiana, United States; 2 MN Epilepsy Group, St Paul, Minnesota, United States; 3 University of Arizona, Tucson, Arizona, United States; 4 University of arizona, Tucson, Arizona, United States

## Abstract

Language use during structured clinical tasks predicts pathological cognitive aging. However, structured tasks reflect only a narrow band of potential communication contexts, which limits the ability to capture cognitive processes manifested in language use under more natural conditions (i.e., minimal constraints). The Electronically Activated Recorder (EAR) makes it possible to sample language from the full ecology of individuals’ interactions. As interactions are cognitively complex, language use in everyday life might be especially sensitive to the integrity of higher-order cognitive processes, including executive functions (EF). Using the EAR and a standard EF battery, we show that EF, particularly working memory, is reflected in analytic (e.g. articles and prepositions), complex (e.g. longer words), and specific (e.g. more numbers) language. The EAR provides first evidence that the words used in daily life reflect the integrity of EF and that reliance on less complex language could reflect WM variability among cognitively healthy adults.

